# Understanding Virus Structure and Dynamics through Molecular Simulations

**DOI:** 10.1021/acs.jctc.3c00116

**Published:** 2023-05-16

**Authors:** Diane L. Lynch, Anna Pavlova, Zixing Fan, James C. Gumbart

**Affiliations:** † School of Physics, Georgia Institute of Technology, Atlanta, Georgia 30332, United States; ‡ Interdisciplinary Bioengineering Graduate Program, Georgia Institute of Technology, Atlanta, Georgia 30332, United States

## Abstract

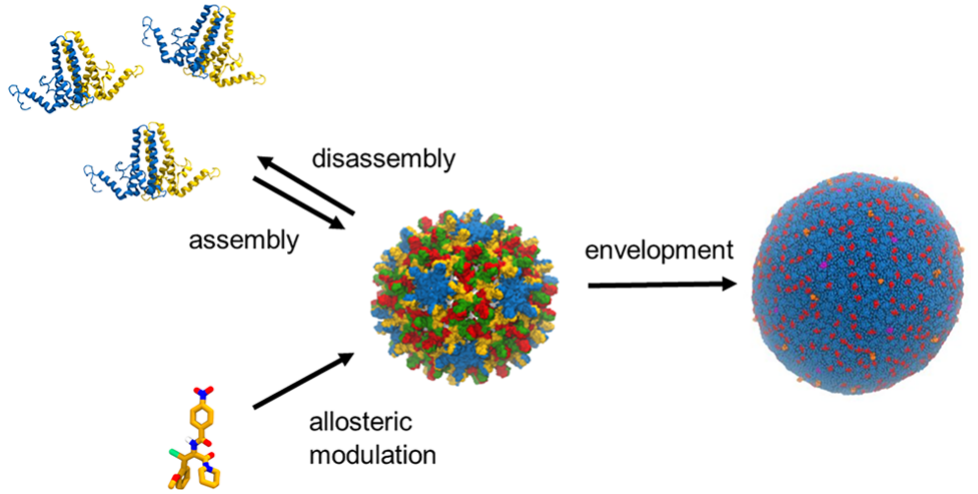

Viral outbreaks remain a serious threat to human and animal populations and motivate the continued development of antiviral drugs and vaccines, which in turn benefits from a detailed understanding of both viral structure and dynamics. While great strides have been made in characterizing these systems experimentally, molecular simulations have proven to be an essential, complementary approach. In this work, we review the contributions of molecular simulations to the understanding of viral structure, functional dynamics, and processes related to the viral life cycle. Approaches ranging from coarse-grained to all-atom representations are discussed, including current efforts at modeling complete viral systems. Overall, this review demonstrates that computational virology plays an essential role in understanding these systems.

## Introduction

Viruses are abundant parasitic particles that produce unprecedented global health and economic threats.^1,2^ A detailed understanding of the various aspects of their life cycle is essential to combat these threats, such as antivirals that adversely modify the viral life cycle to produce noninfectious progeny and vaccines that tune the immune system to fight infection. Moreover, continued efforts are required due to cycles of viral mutations that enhance their evolutionary fitness, whether that be drug resistance^3^ and/or vaccine escape.^4^

Although individual viruses vary in terms of their size, compositional, and structural complexity,^1^ as well as host cell targets, many share similar stages in their life cycles.^5^ Viral particles contain the genomic material required for propagation; however, they must enter and use the host-cell machinery in order to produce additional viral progeny.^6^ Depending on the virus, the viral genome can be circular, double- or single-stranded DNA, as well as double- or single-stranded RNA with either positive or negative polarity. The viral genome is typically encased in a protein shell or capsid. This capsid not only packages the viral genetic material but also protects it from the host immune response. A virus enters the host cell and (i) releases its genome, typically by disassembly of the protective capsid coat, (ii) hijacks the host cell machinery to reverse transcribe and/or translate its genome, (iii) self-assembles the resulting components, and (iv) releases the newly formed viral particles. The production of infectious particles is partially controlled by the proper timing and location of capsid assembly/disassembly and genome packaging. Interfering with any of these stages provides a potential mechanism for antiviral therapeutic applications.^7^ For example, the timing of capsid assembly or aberrant assembly can be modulated/produced by small molecules.^7−10^ In addition to drug-discovery efforts, the self-assembly of capsid proteins into shells has led to applications in medicinal engineering, such as for drug delivery and imaging.^11−13^ Continued development of novel antivirals and engineering applications relies on a detailed knowledge of the structure and function of these viral systems.

Capsids are proteinaceous shells that assemble into a variety of sizes and shapes.^14−16^ In some cases, the capsid self-assembles, while in others, assembly is promoted by cofactors,^17^ scaffolding proteins, or the presence of viral nucleic acids.^14^ Many form a shell with icosahedral symmetry, while others, such as the mature HIV-1 capsid, are pleomorphic, forming a variety of fullerenic cone-shaped structures.^18^ Another example is the Ebola virus nucleocapsid, which forms extended helical shapes with the genetic material intimately associated with the nucleoprotein.^19^ For icosahedral capsids, several capsid proteins form a structural unit that is repeated to make up the complete capsid. Although anomalous icosahedral capsids have been reported,^20^ many icosahedral capsids can be described by Caspar and Klug theory, using the triangulation number (T), which describes how many structural units are required to form each icosahedral face of the capsid.^15^ Moreover, virus capsids can be either enveloped, i.e., encased in a lipid bilayer with embedded structural membrane proteins, or nonenveloped.^14^ The sizes range from tens to hundreds of nanometers.^16^

Studies of viral systems have benefited from the development and application of experimental techniques providing detailed structural information such as cryo-electron microscopy (cryo-EM) and cryo-electron tomography (cryo-ET),^21,22^ in addition to X-ray crystallography, with structures of many complete capsids available.^23^ However, these techniques provide little dynamical information relating to either thermal fluctuations or mechanistic information such as the distribution of intermediates along assembly/dissassembly pathways. In fact, altering assembly is often the focus of antiviral agents, and it is precisely these intermediates that are targeted; however, their transient nature makes experimental study difficult. Solid-state nuclear magnetic resonance (NMR),^24,25^ both in terms of probing events on picosecond to second time scales as well as revealing conformational changes via chemical shift differences, provides complementary structural as well as dynamical information. In addition, experimental advances such as time-resolved atomic force microscopy (AFM)^26^ and time-resolved small-angle X-ray scattering (SAXS) experiments,^27^ which can employ fitting of an ensemble of protein structures to the experimental scattering curves, have made progress not only in identifying classes of highly populated intermediates^28^ but also in visualizing individual pathways as well. In tandem with these experimental approaches, the use of molecular dynamics (MD) simulations in structure-guided applications^29−31^ has yielded atomic resolution for a variety of capsid structures, such as the HIV-1 capsid^18,32^ and the Rous sarcoma virus (RSV) capsid,^33^ becoming an essential tool in their structure determination.

In addition to being a tool for structure refinement, MD is a complementary tool for the study of biomolecular processes, ranging from probing protein–ligand binding at the atomic level to larger-scale simulations, including whole viruses. MD has become indispensable in the interpretation, prediction, and guidance of these experimental studies. With the steady improvement of force fields, hardware, and software, these methods are currently available at increasingly fine levels of spatial and temporal resolution. Although atomistic simulations provide a high level of detail and accuracy, these calculations suffer from large computational demand, necessitating the use of high-performance computing resources, limiting the size and/or time scale of an individual study. In many cases, the resolution of the model is governed by the time scale or spatial extent of the process/system under study. For example, atomistic simulations, which are necessary for a detailed description of protein–ligand interactions central to drug-discovery efforts, are computationally expensive and are limited to short time scales (typically a few microseconds) with limited spatial extent. By introducing approximations to the underlying physical interactions, either by coarse graining (CG) the atomic details or by the application of external forces, events occurring on longer time scales can be investigated. In the case of coarse graining, collections of atoms are combined together, thereby reducing the total number of particles under consideration. Some of these CG methods represent each protein helix by a particle^34^ or otherwise preserve the shape and flexibility of the capsid proteins in order to study a specific virus.^35^ In other cases, rigid models of capsid proteins or capsid subunits have been used.^36−41^ Such models are often used to study general principles of processes related to the viral life cycle, although parameters can be adjusted for specific viruses.^42,43^ Some CG models maintain more details of structures down to the residue level using a specific CG force field like Martini, which typically employs a 4-to-1 atom-to-bead mapping.^44^ Such models could be used to investigate key interactions between components, like the self-assembly process of viral capsids. In addition to reducing the system size, the underlying potential energy surface becomes smoother and thus more rapidly sampled than corresponding atomistic representations, providing an avenue to investigate longer-time scale processes.

**Figure 1:**
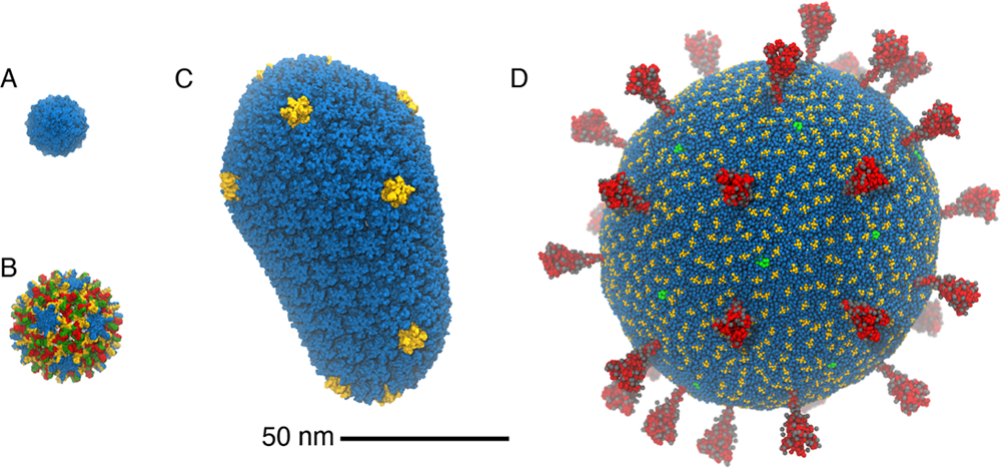
Comparison of some of the viruses simulated with MD, all shown to scale. (A) STMV.^23,45^ (B) HBV.^23,46^ (C) HIV.^32^ (D) SARS-CoV-2.^47^ Images were rendered with VMD.^48^

In the present review, we focus on simulations of enveloped and nonenveloped viral systems with Figure 1 illustrating their size and complexity. Given the system sizes and time scales, various approximations have been employed, and here we provide a description of the computational approaches used to examine viral structure and dynamics, starting with low-granularity coarse graining, moving to finer-grained approaches such as residue-based CG models, and finally moving to atomistic MD. We conclude with a discussion of future prospects for simulating viral systems.

## Low-Granularity Coarse Graining

Various degrees of dimensional reduction can be used in coarse graining. For studies of viral capsids, particle sizes ranging from one particle per capsid subunit to one particle per amino-acid residue have been used. In particular, representing several residues by one CG particle greatly reduces the system size and its degrees of freedom. Although atomistic details are lost through this approach, the advantages are longer simulation times and simplification of simulating processes with an enormous number of degrees of freedom, such as capsid assembly, disassembly, or genome release. In addition, general conclusions about these processes not specific to any virus can be drawn.

Some of the viral capsid simulations used coarse graining to study the dynamics of whole capsids. Arkhipov et al.^35^ used one of the so-called “shape-based” models, in which several beads represent each protein unit. The model fits a number of beads to the protein shape, with a target of ∼200 atoms per bead, taking several model parameters from crystal structures. In order to validate the model, CG simulations were compared to shorter atomistic MD simulations of the satellite tobacco mosaic virus (STMV) capsid, demonstrating that the shape and the size of the atomistic viral particles are well reproduced by the CG simulations.^35^ In the simulations of multiple viruses’ capsids, instability in the absence of nucleic acids was observed, in agreement with experimental data.^35^ The same model was employed for a detailed study of hepatitis B virus (HBV) capsid stability and mechanical properties under experimental AFM indentation.^49,50^ Good agreement between experimental and simulated force response was achieved. The CG model revealed changes in subunit interactions under pressure, not possible to observe in AFM. Specifically, it was shown that subunit bending at the capsid top and bottom, with respect to indentation direction, and small subunit rearrangements around the capsid equator are responsible for structural symmetry changes during capsid deformation.^49^ Through comparison of deformations originating at the three major *T* = 4 symmetry axes, it was shown that capsids are stiffest during deformations originating at the 2-fold axis.^50^

Qiao et al.^34^ developed a model in which each helix of human immunodeficiency virus (HIV) capsid (CA) protein is represented as a single particle in order to preserve the protein shape in their simulations. The authors employed the model in Monte Carlo (MC) simulations to investigate the formation of various capsid assembly intermediates. They showed that of the early intermediates, a trimer of dimers is the most stable, and that these trimers can form the hexameric lattice observed in the assembled immature HIV capsid. However, the assembly of only trimers will eventually cause the formation of flat structures, in contrast to the sharp local curvatures present at specific locations in the HIV capsid. The addition of pentamers of dimers, which are rarely formed and require a particular orientation of the capsid proteins’ N-terminal and C-terminal domains, to the hexameric lattice was necessary to reproduce the expected curvatures.^34^ A study by Wagner et al.^39^ generalized the requirement of pentameric units to induce curvature to other icosahedral viral capsids.

Several studies have used CG approaches with rigid capsid subunits in order to better understand the fundamentals of capsid assembly and other transitions. Notably, Hagan et al.^38^ demonstrated the general importance of unfavorable entropic costs for capsid assembly in order to prevent kinetic traps. Nguyen et al.^36,51^ developed a model in which each capsid protein is represented by several beads in a trapezoidal shape with weak intersubunit interactions. This model was used to study capsid assembly of *T* = 1 and *T* = 3 capsids under various assembly conditions. It was shown that low temperature or high protein concentration can induce capsid misassembly^36^ and that *T* = 3 capsids are more sensitive to these changes in conditions.^51^ The simulations also displayed a nucleation phase followed by an assembly phase in agreement with light scattering experiments.^51,52^ Finally, it was demonstrated that capsid closure can be a slow and energetically unfavorable step due to the entropic costs.^36^ A similar model was used by Rapaport^40,53^ to illustrate that reversibility of subunit binding is crucial for correct assembly, by ensuring sufficient monomer supply and enabling correction of assembly defects.

Perlmutter et al.^37,54^ have modeled assembly of capsid around nucleic acids by describing capsid subunits as pentamers with attractive and repulsive pseudoatoms and attached arginine-binding-motifs (ARMs) of various lengths, which can interact with nucleic acids through electrostatic forces. The model was used to investigate which factors govern the optimal length of the viral genome (*L*_eq_) during capsid assembly. It was shown that capsid size, length of ARM motifs, and increased nucleic-acid base pairing all increased *L*_eq_. Application of parameters from known viruses resulted in good agreement with the experimental *L*_eq_.^37^ The study highlighted the role of the electrostatic forces in capsid assembly and explained the experimentally observed overcharging of capsids, where the negative charge of the genome is larger than the positive charge of the capsid protein. It also showed that nucleic acids can pack to bridge the gaps between positive ARMs even when not directly interacting with them, providing extra stability for the capsids.^37^ The same model was also used to study how salt concentration and subunit interaction strength alter capsid assembly, finding that too weak interactions prevent assembly, while too strong interactions cause misassembly.^54^ Recently, Panahandeh et al.^55^ also studied viral capsid assembly around nucleic acids with a rigid capsid subunit model. It was shown that both the size of the nucleic acid core and mechanical properties of the subunit can determine the preferred capsid triangulation number during the assembly.

A capsid protein model similar to the one used by Perlmutter et al.^37^ was employed by Ruiz-Herrero et al.^56^ to better understand capsid assembly concomitant with budding, which is observed for some viruses e.g., HIV and influenza.^57^ The simulations incorporated membranes and allowed for adsorption of capsid proteins during the assembly.^56^ It was shown that although membrane adsorption increases local protein concentration, which promotes the assembly, the membrane bending required to complete the assembly can have a prohibitive energetic cost. Furthermore, the simulations demonstrate that capsid assembly on a membrane microdomain is more feasible than assembly on a homogeneous membrane due to decreased bending costs of the former.

Assembly of HIV group-specific antigens (Gag) has been studied employing rigid body models of the proteins with multiple binding sites and stochastic reaction-diffusion simulations. Gag consists of several HIV proteins, including CA, and assembles into an immature hexagonal lattice forming interprotein contacts via its CA domain. The lattice has defects characterized by incomplete Gag hexamers at lattice edges, as shown by cryo-ET imaging and analysis.^58^ Qian et al.^59^ investigated Gag assembly, showing that fast on and off rates are required to ensure reasonable assembly rates while avoiding kinetic traps. The role of cellular cofactor IP6, required for Gag assembly, was also explored, demonstrating how rate-limiting nucleation of Gag assembly by IP6 also prevents kinetic trapping. Guo et al.^60^ focused on the mechanism of dimerization between membrane-embedded Gag proteins that also carry HIV reverse transcriptase (Pol). Only 5% of Gag proteins carry Pol (Gag-Pol), and dimerization of two Gag-Pol complexes is the first step in HIV capsid maturation. It was demonstrated that due to the stochastic nature of the assembly, at least some Gag-Pol proteins end up close enough together for spontaneous assembly. In addition, due to lattice defects, Gag and Gag-Pol proteins diffuse easily in the lattice, allowing for additional Gag-Pol pairings.

Several coarse grained studies have modeled genome release in viruses, a process that occurs during infection. A general model by Zandi et al.^61^ showed that both capsid swelling, followed by bursting, and ejection of one of the subunits are possible release mechanisms. Škubník et al.^42^ studied genome release for the family of iflaviridae viruses, namely deformed wing virus (DWV), sacbrood virus (SBV), and slow bee paralysis virus (SBPV), by a combination of CG MD and cryo-EM. The capsids were modeled as pentameric subunits with attractive interactions, and the strength of these interactions was obtained from umbrella sampling with the Martini force field. The capsid of DWV expands at low pH allowing for release of the genome, and simulations of the capsid showed opening of one of the pentrameric capsid units, allowing for release of viral RNA. In contrast, expansion was not observed for SBV and SBPV viruses, and fragmentation of the capsids was seen instead. The different mechanisms could be explained by greater flexibility of the N-termini in DWV, which allows for more flexibility in the capsid shape.^42^ Indelicato et al.^62^ also studied genome release from the human rhinovirus (HRV) capsid, revealing a mechanism similar to the one observed for the DWV, with sequential opening of capsid subunits.

Elastic network models (ENMs) can further simplify the models of viral capsids. In such approaches, the capsid building blocks are modeled as particles that can be interconnected by springs. In spite of their simplicity, many experimental and atomistic or CG MD findings have been reproduced using elastic models. Notably it was shown that ENMs can reproduce surface thermal fluctuations of the capsid observed in atomistic MD and that elastic properties of the capsid can be calculated from an ENM model.^63^ In addition, Panahanden et al.^41^ have used a triangulated elastic sheet model to study the general principles of capsid assembly, focusing on *T* = 3 capsids. Similar to previous findings using more detailed models, it was shown that although formation of hexameric units is most favorable, the formation of rarer pentamers is required to induce curvature in the structures.^34,39^ The model also shows how high protein concentration or strong intersubunit interactions can cause misassembly, in agreement with prior studies.^51,54^ Furthermore, the interplay of hydrophobic interactions and subunit elasticity for correct capsid assembly was highlighted.

**Figure 2:**
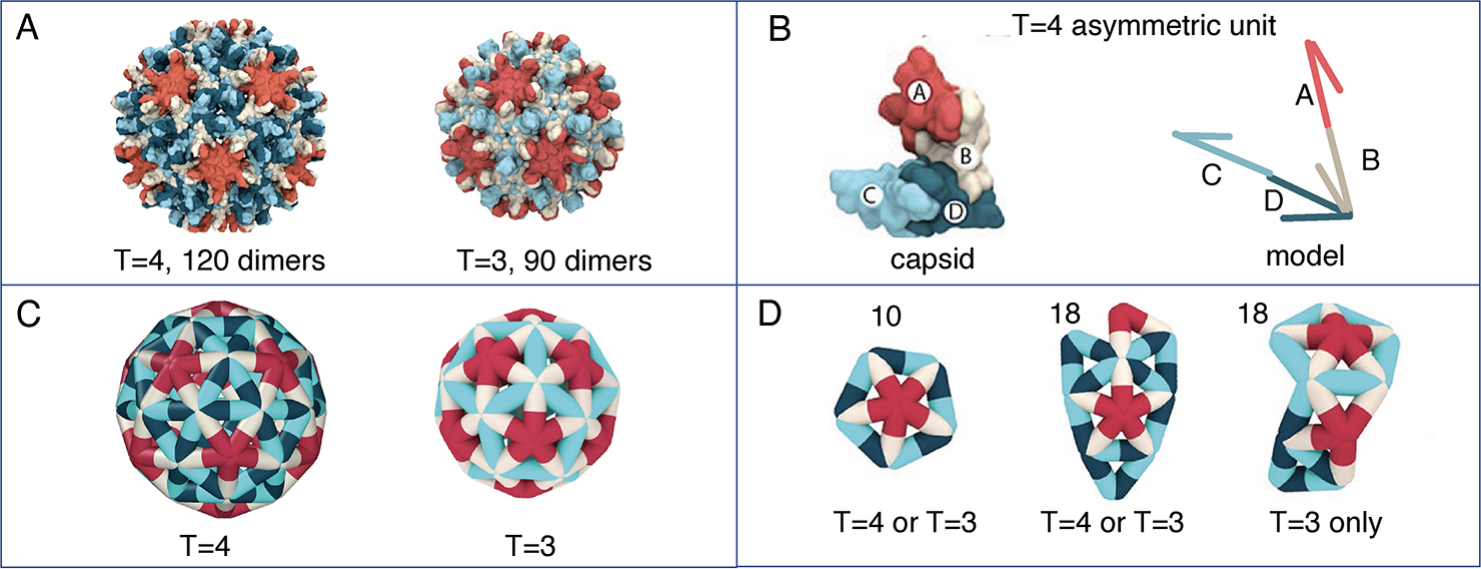
A) Comparison of *T* = 4 and *T* = 3 capsid structures of HBV. B) The structure of the *T* = 4 asymmetric unit of the HBV capsid and the ENM of this unit by Mohajerani et al.^43^ Note the asymmetry of the dimer edges. C) *T* = 4 and *T* = 3 HBV capsids as represented by ENM.^43^ D) Important assembly intermediates on the path to either *T* = 4 or *T* = 3 HBV capsid assembly; the number above indicates the number of dimers in the structure. The left and middle structures can assemble into either a *T* = 4 or *T* = 3 capsid depending on the ratio of CD and CC dimers in the structure, whereas the right structure can only assemble into a *T* = 3 capsid.^43^ Images were adapted from Mojajerani et al.^43^

An elastic sheet model was specifically developed for the HBV capsid by Mohajerani et al.^43^ Many parameters for the model were taken from atomistic simulations of the HBV capsid, and the asymmetry of the capsid units was accounted for.^43^ The *T* = 4 HBV capsid is composed of quasi-equivalent, asymmetric AB and CD dimers of capsid protein with variable strength of contacts, whereas the *T* = 3 capsid is formed by AB and CC dimers (Figure 2A). The elastic model shows that incorporating unit asymmetry and quasi-equivalence of the protein subunits is crucial for capturing several aspects of HBV capsid assembly (Figure 2B,C). It was found that in addition to the specific strength of intersubunit interactions, also investigated by previous studies,^41,54^ the transition energies between quasi-equivalent structures and the different contact energies for these structures are important for correct assembly. The model was validated by comparing simulation outcomes at different assembly conditions to charge detection mass spectrometry (CDMS) experiments. In both experiments and simulations, increased ionic strength resulted in an increased fraction of *T* = 3, whereas higher protein concentrations increased the presence of nonicosahedral structures.^43^ Finally, the paths of *T* = 4 and *T* = 3 assembly were compared, showing that the ratio of quasi-equivalent CC to CD dimers in early assembly intermediates determines the final assembly pathway (Figure 2D).^43^

## Residue-Based Coarse Graining

Residue-based CG models use a bead to represent one or several amino acids, and elastic networks are usually applied to maintain the secondary structures of the proteins. Solvents are usually simplified by using a bead to represent a cluster of solvent molecules. Indeed, the massive amount of solvent is usually an attractive target for simplification. One strategy is the development of hybrid-resolution methods for explicit solvation, which combine different levels of resolution for different parts of the system according to their relevance to the process(es) of interest.

Machado et al.^64^ have developed a hybrid-resolution method, using different levels of coarse graining for solvent with the SIRAH force field. In a traditional fine-grained (FG) model, each water molecule is hydrogen bonded to four neighbors, forming a tetrahedral shape, while the WatFour (WT4) CG model represents a cluster of 11 water molecules with four tetrahedrally connected beads each carrying partial charges. The WatElse (WLS) supra CG (SG) model further reduces the granularity of water by using four connected beads to represent a group of five WT4 clusters, representing 55 FG water molecules in total. In this work, two types of solvation schemes using these water models were developed. The first scheme involved the coexistence of FG/CG/SG, with the inner layer of FG water molecules solvating the protein system surrounded by a second layer of CG water clusters, which was in turn surrounded by a third layer of SC water clusters. The second scheme contained only CG/SG, with the inner solute and CG water surrounded by the outer SG water shell. The layers of different models were well maintained throughout the simulations since only limited mixing was observed at the interfaces between different layers. Several SIRAH models of virus-like particles (VLPs) were simulated with these solvation schemes, and it was observed that the structural descriptors obtained reproduced those from experimental data, including radius of gyration (RGYR), root-mean-square deviations (RMSD), and root-mean square fluctuations (RMSF). These observations indicate that these hybrid-solvation schemes could be potential simplifications of the systems depending on the problems to be investigated.

Martínez et al.^65^ also utilized a multiscale approach to estimate the free energy of viral capsid disassembly. They built a CG model of Triatoma virus (TrV) capsid following the SIRAH force field, which was solvated by the triple-scale FG/CG/SG solvation scheme described above. The CG simulation trajectory was backmapped to atomic snapshots of disassembly paths, which were then used for Poisson–Boltzmann calculations to estimate the free energy of disassembly. Through their calculations, it was determined that the pH of the solvent has a large effect on capsid stability and that the deprotonation of residues within the capsids by alkaline conditions generates an electrostatic repulsion that destabilizes the capsid.

This multiscale solvation scheme could be applied to both MD and CG simulations. Viso et al.^66^ have utilized the FG/CG/SG solvation to study the functions of proton channels on TrV using a combination of MD and CG simulations. In their MD simulations, the atomic capsid was solvated using the triple solvation scheme, and it was found that the channel has a different hydration pattern from the bulk solvent. Quantum mechanical simulations indicated that the flow of water molecules along the channel drives the unidirectional movement of protons, creating an alkaline environment inside the capsid, causing the RNA to unfold, which applies pressure against the capsid. In order to investigate this effect on the capsid stability, the capsid was coarse grained using the SIRAH force field, and chloride ions were placed inside the capsid to mimic the electrostatic repulsion. The CG simulations showed that the imbalance of the internal charge promotes capsid destabilization and disassembly, which releases the viral genome into the host cells.

In addition to solvation, developing an appropriate membrane model is also a challenging problem for multiscale modeling of viruses and VLPs. Several studies^67−69^ have modeled viral envelopes based on cryo-EM structures, which were morphologically consistent with experimental data. Soñora et al.^70^ have also developed an optimized pipeline for building and simulating enveloped VLPs. The protein and lipid models obtained from experimental data were represented by the SIRAH force field. The system was packed using PACKMOL with optimized input options and improved heuristics, and the multiscale CG/SG solvation scheme described above was utilized to solvate the system. CG simulations of the system had good agreement with experimental data in terms of structural descriptors, validating this pipeline for developing multiscale VLP models. More recently, Bryer et al.^71^ and González-Arias et al.^72^ reported the scalable implementation and analysis of a Martini CG model for the HIV-1 liposome, constructed using a realistic, experimentally derived lipid composition asymmetrically distributed over the outer and inner leaflets. Of note, although lipid flip-flop was observed, the lipid asymmetry was maintained. The thickness of the equilibrated vesicle agrees with cholesterol enriched cryo-ET of vesicles. Of particular interest was the observation of transient low-mobility domains occurring only in the outer leaflet of the vesicle. These domains are enriched in cholesterol, sphingomyelin, and phosphatidylcholine. Such regions are implicated in the fusion step of HIV-1 infection.

The self-assembly process of viral capsids is an attractive area of study, but the large number of atoms makes it computationally expensive. Several studies have utilized CG modeling to investigate the self-assembly of HIV capsids. Its major structural protein Gag has a CA domain that plays a pivotal role in its self-assembly. CA is composed of an N-terminal domain (NTD) and a C-terminal domain (CTD) connected by a short linker. Grime et al.^73^ have built CG models to investigate how several features of CA affect the assembly process. CTD and NTD were modeled by two independent stiff ENMs, and the linker connecting CTD and NTD was modeled as a weaker ENM to provide some conformational freedom. It was shown by other studies that the interfacial interactions between adjacent CAs are critical to the assembly process,^74,75^ which were specified to mimic the interactions necessary for assembly. In addition, the conformation of the dimer was incorporated by partitioning CA into two portions: an assembly active portion [*CA*]_+_ and an assembly inactive portion [*CA*]_−_, whose attractive interactions were removed to reflect their incompetence for assembly. The conformational flexibility of the dimers was modeled by randomly reassigning the active portion at a specific switching rate. Through CG simulations, they found that the self-assembly process was sensitive to both CA concentration and molecular crowding, with higher CA concentration as well as crowding favoring the nucleation and growth of lattice structures, consistent with previous studies.^76^ Furthermore, Pak et al.^77^ also conducted CG simulations of HIV capsid assembly incorporating its interactions with other cellular components. They demonstrated the catalytic roles of the plasma membrane and viral RNA during the assembly process through scaffolding for Gag multimerization, suggesting that viral assembly is a concerted process between many factors. Another study by Yu et al.^78^ investigated the interactions between HIV nucleocapsid and TRIM5α, which is an innate immune sensor. It was demonstrated that TRIM5α encages the viral capsid core by forming a hexagonal lattice network and adopting nonhexagonal defects at regions of high curvature, which is consistent with experimental TRIM5α lattice maps. The CG simulations provide a dynamic view of this complicated process, which is complementary to experimental techniques.

Pak et al.^79^ have utilized similar CG models to investigate the mechanisms by which other molecules affect the self-assembly of HIV capsids. Some small molecules, called capsid inhibitors (CIs), can misdirect the self-assembly process of HIV CA proteins. CIs were shown to preferentially bind to the inter-CA pocket formed by the oligomers, such as trimers of dimers (TODs), and stabilize them, biasing the populations of oligomer intermediates.^80^ Therefore, instead of simulating CIs as distinct molecules, their effects were included implicitly by introducing a population of TODs that were CI-bound. An ENM was introduced to maintain the interdimer conformation of the CI-bound TOD. By quantitative analysis of the assembled capsid structures, it was discovered that the CI-bound TODs promoted alternative assembly pathways that led to the formation of noncanonical cores, which was consistent with cryo-EM images of capsid structures bound by the CI GS-CA1.^81^ The formation of noncanonical capsids has two effects on the activities of HIV. First, the inherently large curvature and pleomorphism of these capsids may unsuccessfully enclose the viral genome, limiting its infectivity. Second, these capsids are highly unstable upon entry into host cells, altering the uncoating and trafficking process during infection.

In addition to the effects of CIs, another study by Pak et al.^82^ modeled the effects of salt concentration on the self-assembly of HIV capsids using a “bottom-up” implicit-solvent CG model. In this study, they investigated the effects of inositol hexakisphosphate (IP6), which is present in mammalian cells and known to be an essential assembly cofactor for HIV.^17,83^ Their protein model included the CA domain as well as the spacer peptide 1 (SP1) domain, which is responsible for coordinating Gag oligomerization into lattice structures. The CA/SP1 CG model was constructed based on atomistic MD simulations of an 18-mer of CA/SP1 with IP6 molecules. The electrostatics were represented by a screened Yukawa potential, while monovalent salt concentrations were implicitly modeled by varying the Debye length. Comparing the simulations of low-salt concentration with IP6 versus high-salt concentration only, it was discovered that IP6 accelerated assembly, as well as shifted the morphologies of assembled lattices. Under high-salt concentration without IP6, contiguous hexameric lattice structures were formed, while in the presence of IP6, spherical capsids were generated. Further investigation of free energy revealed that IP6 enhances assembly by reducing the protein–protein association barrier, which causes a kinetically trapped state. Subsequently, fissure-like defects were formed, which resulted in greater curvature of the assembled capsid. The observations agreed with experiments in which spherical capsids formed in the presence of IP6.^17^ However, in vitro assembly experiments in the absence of IP6 generated mature tubes,^17^ while the CG simulations showed relatively flatter lattices, meaning there are still some limitations in this simplified model. Gupta et al.^84^ also discovered that IP6 facilitates conical capsid formation by stabilizing metastable assembly intermediates and thus showed the potential of viral inhibition by targeting IP6 binding sites.

In addition to HIV, other viral systems, like SARS-CoV-2, have also attracted great interest. Yu et al.^47^ constructed a “bottom-up” CG model for the SARS-CoV-2 particles. There are four main structural proteins in SARS-CoV-2, namely the spike (S), membrane (M), nucleocapsid (N), and envelope (E) proteins, whose CG models were constructed separately. The atomic model for each of the proteins was mapped to CG beads using essential dynamics coarse graining (EDCG), while the structures were maintained by heteroelastic network models (hENMs) based on the outcomes of atomistic simulations. The CG model for the membrane envelope consisted of three beads per lipid, and the protein components were embedded to match the envelope protein density from experimental data. By comparing simulations of the CG and atomic models, it was shown that despite some errors, the CG model was able to capture some important features of the atomic model, like radial distribution functions (RDFs) and pair correlations between CG beads. Thus, the trade-off for the improved accuracy is a notable reduction in processes and features accessible compared to the previously described CG simulations. In addition, several studies constructed Martini CG models of SARS-CoV-2 viral particles. Wang et al.^85^ compared Martini models of intact SARS-CoV and SARS-CoV-2 envelopes and found that the structural proteins are uniformly distributed on both envelope membranes. The key difference between the viruses lies in the S proteins, with the intrinsic flexibility of the SARS-CoV-2 S proteins making it easier to recognize and infect the host cells. Pezeshkian et al.^86^ also developed a Martini model of the SARS-CoV-2 envelope by integrating geometric information from multiple pieces of experimental data, and they provided a computational protocol for modeling the envelopes of other coronaviruses.

## All-Atom Molecular Dynamics

Early all-atom MD studies of viral capsids employed rotationally symmetric boundary conditions, thereby reducing their computational demand,^87−89^ although asymmetric motion in the capsid was not captured. Multiscale approaches, e.g., coupling short-time atomistic MD with an extrapolation procedure^90,91^ or multiscale factorization,^92^ have also been applied to viral systems. Atomistic MD simulations prior to 2012 have been limited to a few million atoms on time scales significantly less than ∼1 μs. However, in the past decade, these studies have been extended to larger systems and longer trajectories; here we focus on simulations of complete capsids or complete viral envelopes. Table 1 provides a concise summary of these simulations, including the approximate atom counts and simulation lengths.

**Table 1:** Summary of All-Atom Simulations of Viral Systems

System	Atom Count (10^6^)	Simulation Length (μs)^a^	Year
STMV^45^	1	0.04	2006
SBMV^93,94^	4.5	0.1,0.15	2009,2010
HPV-16^95^	4	0.01	2011
STNV^96^	1.2	4	2012
Poliovirus^97^	3–4	0.02	2012
HIV-1^32^	64	0.2	2013
Poliovirus^98^	6.5	0.2	2014
HBV^8^	6	0.2	2016
PCV2^99,100^	1.9	0.05,0.02	2017
HIV-1^101^	64	1.2	2017
HBV^46^	6	1.1	2018
MS2^102^	3.5	0.05	2019
Zika/dengue^103^	12	0.12	2020^b^
Influenza A^104^	160	0.12	2020
HBV^105^	6	1	2021
HBV^106^	6.5	0.6	2021
HBV^107^	8	0.2	2021
SARS-CoV-2^108^	305	0.2	2021
HIV-1^109^	76	0.01	2021
RSV^33^	1.8	0.02	2021
MVM^110^	3	2.0	2022
Influenza A^111^	160	0.9	2022
HIV-1^18^	44–76	1.6	2022
SARS-CoV-2/aerosol^112^	1000	0.0024	2023

a The simulation length represents the approximate total of all replicas and models.

b This study used a united-atom model, with polar hydrogens retained.

In 2006, Freddolino et al.^45^ carried out one of the first simulations of a fully hydrated, all-atom, full virus particle for STMV (∼1M atoms, ∼40 ns). STMV is small (∼17 nm diameter), and the simulations, with and without a model for the viral genome, illustrated that without the incorporation of the RNA, severe instability of the capsid resulted. In addition, not only was the loss of icosahedral symmetry observed, but also global correlated/anticorrelated motions were reported, demonstrating the benefit of including the entire capsid, rather than imposing icosahedral symmetry.

Simulations of increasingly larger capsids for longer time scales followed. Zink and Grubmüller^93,94^ reported the mechanical response of southern bean mosaic virus (∼4.5M atoms, ∼250 ns), with and without Ca^2+^ ions, to nanoindentation via force-probe MD, revealing that the capsid behaves elastically prior to rupture. Given that the capsid must distort/rupture in order to expel its cargo, mechanical properties are of interest for unraveling the mechanism of genome release. Joshi et al.^95^ simulated subunits and the *T* = 1 capsid of human papillomavirus-16 VLP (∼4M atoms, ∼10 ns) and reported reduced fluctuations in the antigentic L1 loops of the capsid. Pentamers are known to have reduced immunogenicity relative to the complete VLP, and these results suggest a correlation between reduced flexibility and immune response. Roberts et al.^97^ (∼4M atoms, ∼20 ns) and Andoh et al.^98^ (∼6.5M atoms, ∼200 ns) reported MD simulations of poliovirus, with the former exploring stability of an amphipathic N-terminal helix, unobserved in structural studies. Andoh et al.^98^ explored microscopic properties of the capsid and reported rapid water exchange across the surface. At room temperature, the capsid is tolerant to applied pressure. The exchange rates reported would result in rapid pressure equilibration across the capsid shell, thus explaining the observed pressure effect. Tarasova et al.^99,100^ and Farafonov and Nerukh^102^ have applied MD to the capsids of PCV2 (∼1.9M atoms, ∼70 ns) and MS2 bacteriophage (∼3.5M atoms, ∼50 ns), respectively. Both the PCV2 and MS2 simulations revealed a charge neutralizing Cl^–^ layer inside the capsid structure. Without this layer, the PCV2 capsid is unstable, suggesting that electrostatics plays an important role in maintaining its structural integrity, while the MS2 simulations indicate that capsid pores provide pathways for ion and water flux.

Larsson et al.^96^ applied microsecond-time scale MD to the capsid of satellite tobacco necrosis virus (STNV, ∼1.2M atoms). STNV has three types of Ca^2+^ binding sites, and release of Ca^2+^ has been associated with swelling of the capsid, a process implicated in genome release. With Ca^2+^ ions removed, swelling of the capsid was observed, and regions near acidic residues with high RMSF were identified, suggesting that these areas play a role in the initial dissolution of the capsid. In addition, microsecond-time scale MD has been applied to the HBV capsid by Hadden et al.^46^ (∼6M atoms), where significant flexibility and asymmetric global motion were observed, providing insight into mechanical properties that may be relevant to the virus life cycle. The authors show that sample heterogeneity, resulting from capsid flexibility, sets a limit on the cryo-EM resolution. Moreover, water-exchange rates indicate that rapid pressure equilibration is possible upon mechanical distortions, with ions also undergoing exchange. Importantly, subsequent solid state NMR experiments^113^ were performed for the HBV capsid. Retroactive analysis of this 1-μs HBV trajectory reveals that for motion on the nanosecond time scale, there is good agreement with these measurements. Fujimoto et al.^107^ modeled the pgRNA-containing HBV capsid (∼8M atoms, ∼200 ns). The computed electric field, produced by the solvated capsid, revealed regions near the spike tips where a negative point charge would experience a repulsive force, due to acidic residues located there. However, between the spike tips, the force exerted on a negative test charge is either negligible or attractive. These results suggested that negatively charged species, such as nucleotides necessary for reverse transcription, approach the capsid avoiding the tips and then find and enter it via pores. In addition, permeation rates were obtained for the cations (K^+^, Na^+^) and anions (Cl^–^), revealing ionic selectivity, with the Cl^–^ rates being approximately an order of magnitude smaller than those of the cations, in accord with earlier apo simulations.^46^ Heat-induced structural changes for the parvovirus minute virus of mice (∼3M atoms, ∼2 μs) were studied by Pathak and Bandyopadhyay.^110^ A breathing transition, i.e., a temperature-induced structural change, was observed at 318 K. This motion was further analyzed using atomic stress analysis, which indicated regions of maximal stress distant from the capsid pores and in good agreement with hydrogen/deuterium exchange mass spectrometry results.

**Figure 3:**
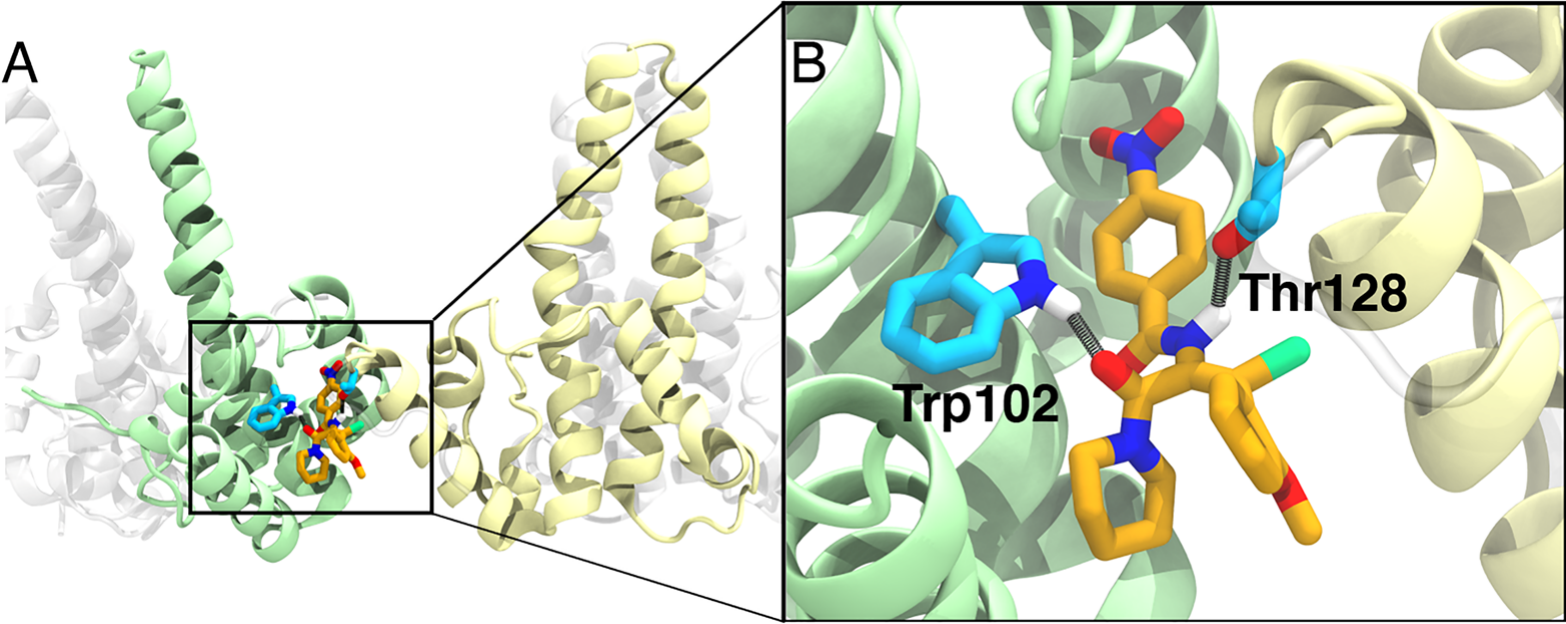
Binding pocket of AT-130. A) AT-130 binds at an interdimer interface of HBV. HBV’s core protein in the tetrameric unit is rendered in a ribbon, with the interdimer-contact monomers colored green and yellow. B) Illustration of hydrogen bonding between AT-130 and Trp102 and Thr128 of the core protein. Carbons are rendered in orange (AT-130) and cyan (HBV), while nitrogens, oxygens, and bromine are colored blue, red, and bright green, respectively. The structure was taken from Pavlova et al.^10^

Capsid assembly is an essential step in the viral life cycle; as such, it is an attractive target for drug development. For example, core protein allosteric modulators (CpAMs) have been shown to alter the timing and organization of HBV capsid assembly, with several of these modulators currently in clinical trials.^114^ Given this interest in capsid assembly modulation, the HBV simulations of Hadden et al.^46^ were analyzed to provide an explanation for the effects of T109 mutations (T109M and T109I) in antiviral resistance, suggesting that these larger hydrophobic residues may occlude the binding pocket.^115^ Perilla and Schulten^8^ reported 100 ns simulations of apo and HAP1-bound HBV (∼6M atoms). HAP1 is a misdirector, producing aberrant assembled structures. Their simulations indicated an enhancement of quaternary structural arrangements seen in the crystal structure upon HAP1 binding and suggested a global structural perturbation due to the ligand. Moreover, Pérez-Segura et al.^105^ reported simulations in which the CpAM AT-130, an assembly accelerator, was included (∼6M atoms, 1 μs). Applying network analysis to the apo and AT-130-bound systems revealed alterations in communication patterns as a result of AT-130 binding. The HBV core protein contains several α-helices, two of which (helices 3 and 4) protrude from the capsid surface forming a spike. Features associated with efficient assembly, namely recovery of the spike tips’ secondary structure, smaller bending angles of helix 4, and a well-ordered interface, were observed and attributed to the presence of AT-130. In Figure 3, the binding pocket of AT-130 is displayed, illustrating hydrogen-bonding interactions with specific capsid residues (Figure 3B), thus highlighting the need for all-atom representations to capture these directional electrostatic interactions.

The above simulations of HBV have provided a detailed description of the equilibrium properties of its capsid as well as a CpAM-bound state on the microsecond time scale. Recently, Ghemei et al.^106^ have applied atomistic MD studies to an early stage of HBV capsid dissassembly by employing mechanical stress via an external isotropic force acting to expand the capsid. Starting from an equilibrated capsid (∼6.5M, ∼0.6 μs), 20 independent trajectories using this applied force were performed, with initial stages of dissassembly observed to take place in just 6 ns. These simulations indicated that the capsid protein dimers are quite stable, but cracks first appear primarily at the hexameric interfaces and less often at pentameric ones. Lastly, specific residues or hotspots with weak interdimer interactions were identified. These residues are highly conserved and prevent an overstabilization of the interdimer interface, which would retard disassembly. Taken together, these simulations provide a detailed picture of the first steps in the release of HBV genomic material.

Viral capsid structures beyond those with icosahedral symmetry have been investigated. The HIV-1 capsid has been simulated using all-atom MD in a series of ground-breaking studies, particularly in terms of system size.^18,32,101^ Zhao et al.^32^ reported the first all-atom model of the mature capsid (64M atoms, 200 ns) by combining cryo-EM maps and MD flexible fitting.^29^ The fully solvated model was compared to an immature retrovirus structure, suggesting that large conformational changes occur upon maturation. Moreover, these simulations^32^ were used in additional studies. For example, Cyclophilin A (CypA) is a host factor involved in regulating HIV-1, and mutations in the HIV-1 loop which binds CypA are known to reduce infectivity. Additional analysis revealed a reduction in flexibility of the CypA binding loop upon mutation, suggesting an allosteric mechanism of regulation^116^ and that pentamers are more rigid than hexamers,^117^ both in good agreement with NMR measurements. Using the model of Zhao et al.,^32^ Perilla and Schulten^101^ reported a 1.2-μs simulation of the fully solvated HIV-1 capsid. They were able to address several atomic-scale properties of the capsid, including the translocation of water and ions, Na^+^ and Cl^–^ ion-binding-site distributions, and an equivalence of the electrostatic potential between the interior and exterior of the capsid. Globally, they observed mechanical oscillations that spread across the surface of the capsid, revealing collective correlated and anticorrelated motions. In addition, an all-atom fullerenic model of the complete native HIV-1 capsid has been constructed using refined hexameric and pentameric units,^109^ and a short unrestrained simulation (76M, ∼0.01 μs) was performed, providing an updated, realistic model.

Recently, Yu et al.^18^ have generated a series of mature HIV-1 capsid models containing either (i) water, (ii) ribonucleoprotein (RNP), (iii) bound IP6 cofactors, or (iv) both IP6 and RNP. The systems ranged from 44M-76M atoms and were simulated for a collective 1.6 μs, demonstrating increased strain in the capsid upon binding of the cofactors, with regions of high strain stretching across its surface. Distinct atomic-level conformational adjustments were observed at the pentameric interfaces in the capsid, such as an increase in the size of the pores and an increase in the local curvature, while hexameric ones were largely unaffected. Moreover, a CG model for rupture displayed the formation of cracks along these high-strain regions, matching cryo-ET of HIV-1 obtained during reverse transcription. Overall, this study indicates that mechanical properties of the HIV-1 capsid can be tuned by cofactor binding and suggests that these properties could be modulated to disrupt the viral life cycle, providing a structural basis for therapeutic interventions.

A high-resolution structural model of the *T* = 1 capsid of Rous sarcoma virus (RSV) has been generated using an integrated solid-state NMR-guided MDFF approach (∼1.8M, 20 ns).^33^ Principal component analysis of the individual monomers from the tubular and capsid assemblies revealed that rotation about a flexible N-terminal/C-terminal interdomain linker is associated with the curvature modulation observed in tubular lattices versus capsid assemblies.

Flaviviridae, such as Zika and dengue, share a similar glycoprotein shell structure, composed of E and M proteins arranged in an icosahedron, with the E protein surface exposed. However, at 37 °C, the Zika shell remains intact, while the dengue shell displays evidence of instability. The icosahedral glycoprotein shell is composed of 30 rafts, with each raft composed of three E protein homodimers. The united-atom work of Pindi et al.^103^ comparing the Zika and dengue shells (∼12M, ∼0.12 μs) revealed that at 37 °C, the dengue shell displays looser raft–raft interactions, with fewer contacts and polar interactions than those in the Zika shell. Moreover, they observed holes forming at the three- and five-fold vertices in the dengue shell. Taken together this work provides a molecular level explanation of the differential stability of these viruses.

Among the largest systems studied to date with all-atom MD is the viral envelope of influenza A by Amaro and co-workers,^104,111^ which included the glycoproteins hemagglutinin (HA) and neuraminidase (NA), the proton channel M2, and a phospholipid bilayer. NA cleaves sialic acid residues from host-cell receptors, thereby assisting in viral particle release. While the NA catalytic site (primary or 1° site) has been the focus of drug design, a secondary site (2°) has also been identified. Of interest, the 1° site is closed in the crystal structure, while the role of the 2° site is not entirely understood. MD simulations^104^ (∼160M atoms, ∼0.12 μs) were used to address the opening, via flexible loops, of the 1° catalytic site and to kinetically characterize loop opening/closing via a Markov State Model (MSM). In addition, a pathway lined with basic residues from the solvent-exposed 2° site to the 1° site was identified, suggesting a novel substrate-binding mechanism. More recently,^111^ ∼0.5-μs simulations were performed for the fully glycosylated influenza A model as well as a second strain. This study revealed three major conformational transitions of the NA and HA proteins and identified novel epitopes, e.g., on the underside of the NA head, as well as transient exposure of a highly conserved cryptic antibody binding site upon a HA breathing motion. Both of these sites have been characterized experimentally and highlight the role MD can play in characterizing these interactions at the molecular level. Similar to their earlier work,^104^ MSMs were generated providing a kinetic characterization of these large-scale protein motions. Lastly, this study illustrated dynamic networks of HA/NA interactions across the viral surface, highlighting the role of the glycans in a realistic viral environment.

## Outlook

Significant progress in understanding viral structure and dynamics has been achieved over the past two decades. Here we have summarized computational contributions to this understanding. Practically all models developed at all three resolution scales covered here rely on both the input of experimental data during their design as well as comparison to available experimental data for validation. For example, in the case of assembly simulations, the observed kinetics and their dependence on subunit concentration or temperature can be compared to, e.g., SAXS or other light scattering techniques.^36,41,59^ The different outcomes due to assembly conditions in the simulations can also be compared to CDMS experiments.^43^ In the case of residue-based CG simulations of capsid assembly, the observed structures are usually compared with the observations by cryo-EM^47,73,78,79^ or cryo-ET.^77^ Fluorescence techniques like total internal reflection fluorescence (TIRF) also enable validation against in vitro experiments.^77^ Finally, all-atom studies reported good accord with hydrogen–deuterium mass spectroscopy,^110^ SAXS,^106^ and magic angle spinning solid-state NMR^113,116,117^ data. In addition, the recent demonstration of antigenic sites on the influenza A surface glycoproteins is in accord with structural studies for that system,^111^ illustrating the predictive power of these simulations. Moreover, for coarse-grained models, validation has been performed by comparing the outcomes to all-atom simulations.^35,64^ Overall, such comparisons generate confidence in simulation results for each model’s spatial and temporal regimes.

Undoubtedly this work will continue, for example, in low-granularity coarse-grained methods, where the focus has been on improving model accuracy, often by fitting model parameters from less coarse-grained or even atomistic simulations.^42,43^ Retaining the original protein shape is important in CG simulations, and software to automate the development of shape-based models with particle parameters from atomistic simulations has been recently developed.^118^ Similarly, residue-level CG models are also constructed from experimental data or atomic models in a bottom-up way, which are system-dependent. Alternatively, top-down approaches, which utilize generalized structural patterns and are thus more transferable, are an open field to be explored for future model designs.^119^

As we enter the exascale computing era,^120^ we can expect longer, more detailed simulations to be performed with all-atom descriptions of complete systems becoming more common. For example, two of the largest all-atom studies to date are those of Casalino et al.^108^ for the complete SARS-CoV-2 viral envelope (305M atoms, 0.2 μs) and of Dommer et al.^112^ for a SARS-CoV-2 respiratory aerosol model (1B atoms, 0.0024 μs). It is particularly interesting to note the difficulty in building and equilibrating these systems, such that new tools and protocols are required, which are currently being developed.^72,121−124^ González-Arias et al.^72^ describe scalable analysis for the CG HIV-1 lipid envelope on the Frontera supercomputer. In addition to equilibrating the vesicle using the Martini force field, backmapping to an all-atom vesicle model and minimization was performed, yielding a system with 280-million atoms. Moreover, while a few studies have included genomic material in viral simulations, efforts to do so have been hampered primarily due to a lack of structural information. Viral systems will undoubtedly benefit from integrated experimental and modeling approaches, such that the simulation of complete, enveloped viral systems is likely to become the focus of computational virology.
